# Mathematical model estimation of dengue fever transmission risk from Southeast and South Asia into Japan between 2016 and 2018

**DOI:** 10.1265/ehpm.22-00267

**Published:** 2023-09-09

**Authors:** Ken Sakamoto, Takenori Yamauchi, Akatsuki Kokaze

**Affiliations:** Department of Hygiene, Public Health and Preventive Medicine, School of Medicine, Showa University, Tokyo, Japan

**Keywords:** Dengue, Mathematical model, Importation, Autochthonous, Japan

## Abstract

**Background:**

Dengue fever is a viral infection transmitted to humans through the bite of a mosquito infected with the dengue virus. Dengue is one of the most common infectious diseases in the world, and its incidence is rapidly increasing. We estimated the risk of dengue importation from endemic countries to Japan and the transmission risk within Japan using data collected between 2016 and 2018.

**Methods:**

We conducted simulations that included the number of reported dengue infections and travelers per month in ten countries in Southeast and South Asia.

**Results:**

The estimated importation risks for Japanese returnees and international travelers from each of the ten endemic countries was approximately 1.0 every month from 2016 to 2018. The autochthonous transmission risk in Japan from any target country was 1.0 from June to September yearly. The estimated number of Japanese dengue cases returning to Japan is approximately 25 times higher than that of imported cases reported in Japan.

**Conclusions:**

The risk of dengue importation into Japan can be sufficiently high. Attention should be paid to autochthonous transmission spread between June and September when mosquitoes are active in Japan. Estimates of seasonal risk variation from each dengue virus-endemic country can be used to inform preventive and control measures for dengue in Japan.

**Supplementary information:**

The online version contains supplementary material available at https://doi.org/10.1265/ehpm.22-00267.

## Background

Dengue fever (DF) is a viral infection transmitted to humans via mosquito bites infected with dengue virus (DENV). The primary vectors used were *Aedes aegypti* and *Ae. albopictus*. It is one of the most common infectious diseases in the world, and its incidence is rapidly increasing worldwide, with an estimated 100–400 million infections annually [1]. Although more than 80% of infected individuals are mildly ill and asymptomatic, cases of severe illness and death exist. DENV has four antigenic subtypes, and although it acquires lifelong immunity to a serotype once infected, cross-immunity to different serotypes disappears over time; therefore, one may be infected with other types. A second infection increases the risk of developing severe dengue hemorrhagic fever (DHF) [2, 3].

In Japan, dengue autochthonous transmission had not been reported for approximately 70 years since western Japan’s report in the early 1940s [4] but was reported in 2014 [5] and 2019 [6]. Currently, the DENV vector in Japan is *Ae. albopictus*, but *Ae. aegypti* has not yet been established. However, in recent years, larvae and adults of *Ae. aegypti* has been collected around Narita International Airport and Tokyo International Airport [7], and given the increase in air travel and rising temperatures, *Ae. aegypti* could establish in Japan in the future. Conversely, *Ae. albopictus* originally existed in Japan, but in recent years, its habitat has gradually expanded to the north [8, 9].

In Japan, dengue is classified as a Class IV infectious disease under the Infectious Disease Control Law, and all physicians are required to notify the government through local public health centers at the time of diagnosis [10]. Physicians were required to report demographic information, clinical history, and exposure history for all patients who met DF/DHF definition. However, a review of seroprevalence in Southeast Asia suggests a large discrepancy between reported cases and imported cases [11]. Moreover, DF incidence among Japanese travelers varies depending on country and season [12].

In this study, we conducted simulations using the number of reported cases and travelers per month in ten countries in Southeast and South Asia (SEA/SA) to estimate dengue importation risk from endemic countries to Japan and autochthonous transmission risk within Japan using data from 2016 to 2018. To the best of our knowledge, this is the first report of a simulated estimation of the number of imported cases per dengue-endemic country and per month using Japan as the model.

## Methods

### Target dengue-endemic countries

The ten countries covered in this study were SEA/SA (Cambodia, India, Indonesia, Malaysia, Myanmar, the Philippines, Singapore, Sri Lanka, Thailand, and Vietnam). These countries were selected because the number of reported cases per month in each country was available for 2016–2018. They were 10 of the top 11 countries in the number of imported dengue cases reported in Japan (data were not available for Bangladesh, which ranked 10th) and accounted for 84% of all reports between 2016 and 2018.

### Expansion factors

The expansion factor (EF) is a coefficient used to estimate the total number of dengue cases in each country to consider healthcare policy [13]. The actual number of cases was estimated using this factor multiplied by the number of reported cases in each country’s dengue surveillance system, which is usually underreported. The EF parameters were treated as distributions based on previous reports [13–15] (Table 1).

**Table 1:** The expansion factors by country.

**Country**	Distribution*	**Distribution parameters**	**Values**	**Reference No.**
Cambodia	PERT	(Mode, Min, Max)	(12.9, 3.9, 29.3)	13
India	Triangular	(Mode, Min, Max)	(282, 176, 717)	15
Indonesia	PERT	(Mode, Min, Max)	(7.6, 7.1, 9.9)	13
Malaysia	PERT	(Mode, Min, Max)	(3.8, 2.5, 6.2)	13
Myanmar	Normal	(Mean, SD)	(16.2, 8.9)	13
Philippines	Normal	(Mean, SD)	(7.0, 0.5)	13
Singapore	PERT	(Mode, Min, Max)	(4.1, 1.0, 4.9)	13
Sri Lanka	PERT	(Mode, Min, Max)	(21.5, 8.3, 45.6)	14
Thailand	PERT	(Mode, Min, Max)	(8.5, 8.0, 12.5)	13
Vietnam	PERT	(Mode, Min, Max)	(5.8, 5.4, 6.7)	13

Max: maximum, Min: minimum, PERT: program evaluation and review technique, SD: standard deviation.

*The PERT and triangular distributions were approximated using a beta distribution, respectively [13, 14].

### Number of reported dengue cases per month in SEA/SA (2016–2018)

The number of reported cases in Cambodia was obtained from Cousien et al. [16] and the World Health Organization (WHO) website [17]. For India, the number for each month of 2016–2018 was unavailable; the ratio of cases per month was estimated based on the average number of tested patients and the percentage of dengue positivity per month for 2014–2017 from Murhekar et al. [18]. The number of cases reported for 2016–2018 was allocated [19] by that ratio. The number of reported dengue cases in Indonesia for 2016 was obtained directly from Husnayain et al. [20]; for 2017 and 2018, we averaged the number of reports in each month of 2012–2016 reported by Husnayain et al. [20] and allocated them as a percentage of the annual number of reports obtained from a news article [21]. Information was obtained from the WHO websites for Malaysia, the Philippines, and Vietnam [17]. Information on Myanmar was obtained from the WHO and news articles [22–24]. The percentage of each month was calculated from the total number of reports in each month for 2010–2015, and the annual number of reports in 2016–2018 was allocated to each month using that percentage. Information for Singapore [25] and Thailand [26] was obtained from their respective government websites.

When only graphs were available and no numerical information, numerical data were obtained using the WebPlotDigitizer [27]. In the case of weekly reporting numbers (usually reported in 52 weeks/year), January 1 was used as the start date and was assigned to each month daily. A leap year was also considered in this study.

### Number of monthly imported dengue cases among Japanese

Information was obtained from the National Institute of Infectious Diseases website [28].

### Number of Japanese departing from Japan per month

Information on travel to Cambodia, Malaysia, the Philippines, Singapore, Thailand, and Vietnam was obtained from the JTB Tourism Research & Consulting Co. website [29]. For India, we extrapolated the percentage of Japanese travelers for each month from the 2016 Nielsen survey results [30] and the annual number of Japanese departures for 2016–2018 from the Japan National Tourism Organization (JNTO) website [31]. Data for Indonesia from 2016 and 2017 were obtained from the Indonesian Tourism Officer’s official website [32]. In 2018, the annual number of Japanese visitors to Indonesia was obtained from the JNTO website [31], and the number of visitors for each month was extrapolated using the percentage of monthly data for Bali obtained from JTB [29]. For Myanmar, data on the monthly percentage of Japanese arrivals at the three international airports were obtained from the relevant government website [33] and extrapolated using JNTO’s annual number of Japanese visitors [31]. Information about Sri Lanka was obtained from a published tourism and information website [34].

### Number of international visitors to Japan per country per month

Information on visitors from SEA/SA was obtained from JNTO [31].

### Population trends by year for each country

The population by year between 2016 and 2018 for each country was obtained from the World Bank open data [35].

### Japanese travelers’ average length of stay in SEA/SA countries

The average length of stays in Cambodia [36], India [37], Indonesia [38], Myanmar [39], Philippines [40], Singapore [41], and Sri Lanka [42] were obtained from government-related public documents. Information on Malaysia [43, 44], Thailand [45], and Vietnam [46] was obtained from publicly available websites and documents.

### SEA/SA nationals’ average length of stay in Japan

The information is obtained from JNTO [31].

### Temperature conditions in Japan

We used the monthly average temperature in Tokyo published on the Japan Meteorological Agency website [47] as the temperature in Japan.

### Dengue importation (*P_IMPORT_*) and dengue autochthonous transmission risks in Japan (*P_AUTO_*)

We defined *P_IMPORT_* as the probability that at least one DENV-infected traveler is flying from SEA/SA into Japan and is infectious upon arrival in Japan, and *P_AUTO_* as the monthly probability that at least one person in Japan has autochthonous transmission by DENV brought from SEA/SA. The calculation method for each is shown in the Supplementary Document. As model’s application situation, *P_IMPORT_* assumed that all travelers from Japan and the endemic countries would return to their countries within the same month. Moreover, the exposure risk was the same for both Japanese travelers and residents of the endemic countries. *P_AUTO_* used the average temperature in Tokyo for Japan. Both symptomatic and asymptomatic infections were included in the expected values calculated. Human-mosquito contact was assumed to be uniform.

### Data organization and statistical analysis

Simulations were run, and graphs created using Microsoft® Excel® for Microsoft 365 MSO (version 2207 build 16.0.15427.20182) 64-bit. R version 4.2.0 (2022-04-22 ucrt) was used to create boxplots and calculate the Pearson product-moment correlation coefficient and *P*-value. The correlation coefficient (*r*-value) of 0.2 < *r* ≤ 0.4 was considered “weakly correlated,” 0.4 < *r* ≤ 0.7 was considered “moderately correlated,” and 0.7 < *r* was considered “strongly correlated” (the significance level was set at 5%). Since multiple parameters were used in our simulation, outliers were expected to be very large. Therefore, outliers were removed using 95% data interval (DI).

## Results

**Fig. 1:**
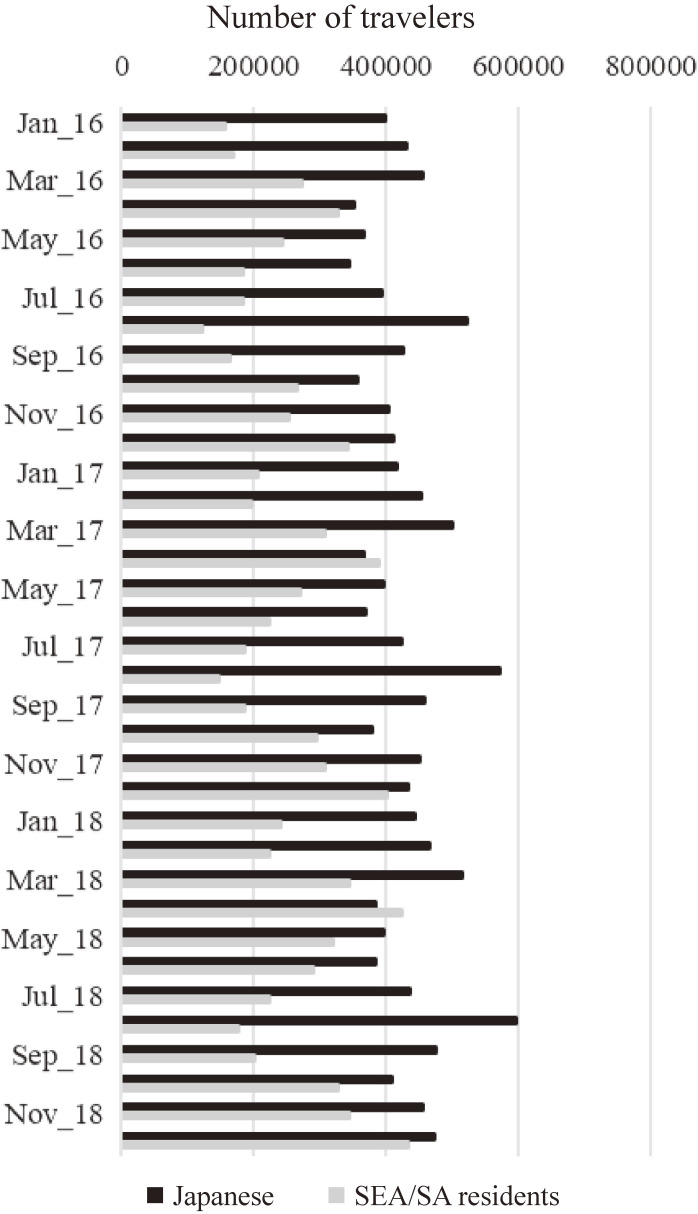
Total travelers between Japan and ten SEA/SA countries, 2016–2018.

Between 2016 and 2018, total Japanese travelers to SEA/SA countries increased annually to 4.86 million (2016), 5.22 million (2017), and 5.44 million (2018). This represented 28.3%–29.2% of all Japanese departures during the same period. Travelers from SEA/SA countries to Japan increased in number over the years to 2.69 million (2016), 3.12 million (2017), and 3.56 million (2018). This was 10.9%–11.4% of all inbound travelers during the same period (Fig. 1, Supplementary Table).

### Dengue importation risk into Japan

**Fig. 2:**
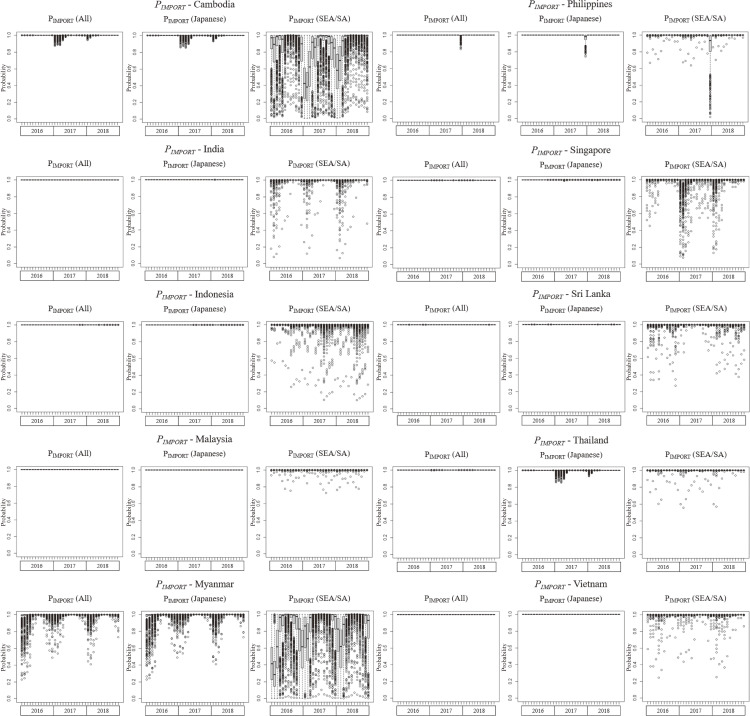
Dengue importation risk into Japan (*P_IMPORT_*). The distribution for each month is shown as a box plot.

We examined the monthly dengue importation risk in Japan from 10 SEA/SA countries (Fig. 2). For all countries, the importation risk for the sum of Japanese returnees and SEA/SA travelers to Japan (*P_IMPORT_* [all]) and Japanese returnees (*P_IMPORT_* [Japanese]) was approximately 1.0. For SEA/SA visitors to Japan (*P_IMPORT_* [SEA/SA]), the value was approximately 1.0, although it was not as high as *P_IMPORT_* (all) and *P_IMPORT_* (Japanese).

### Dengue autochthonous transmission risk in Japan

**Fig. 3:**
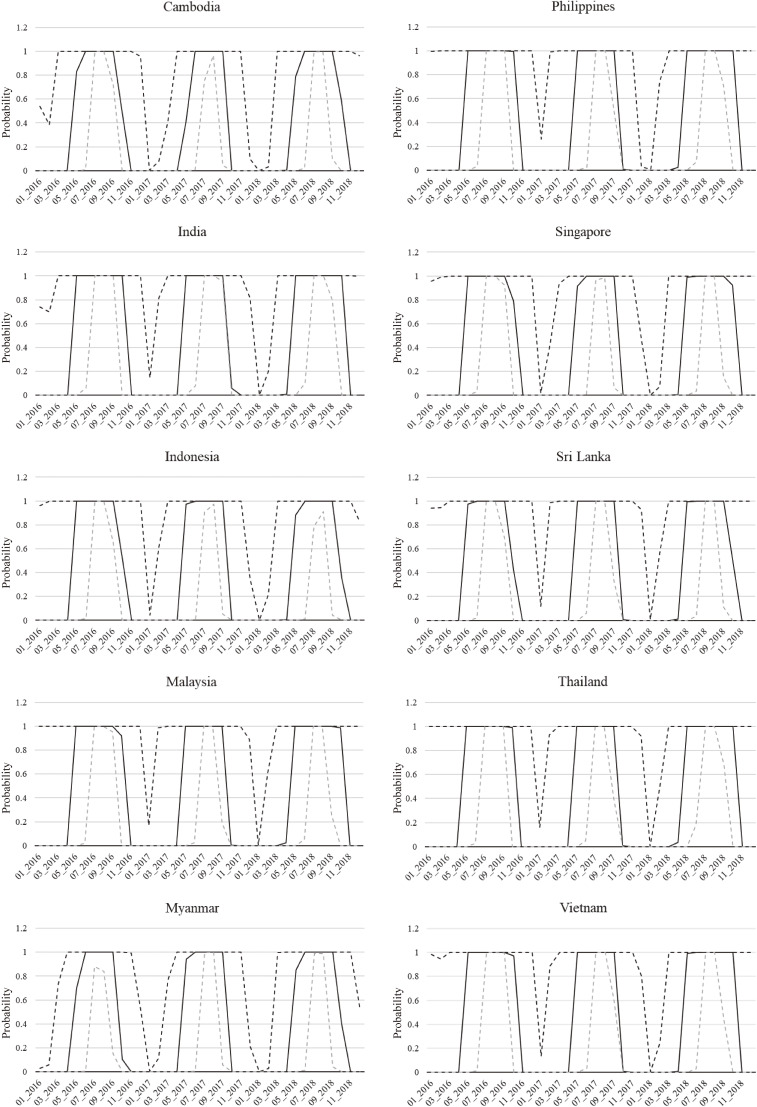
Dengue autochthonous transmission risk in Japan (*P_AUTO_*). The solid line for each country is the median and the dashed lines represent the 95% DI.

The monthly autochthonous transmission risk (*P_AUTO_*) in Japan by international travelers from 10 SEA/SA countries and Japanese returning to Japan was calculated for each SEA/SA country (Fig. 3). As a result, it was estimated that the autochthonous transmission risk in Japan from any SEA/SA country increases to 1.0 under the condition of an average temperature ≥22 °C from June to September in Tokyo.

### Estimated symptomatic Japanese cases and actual imported cases reported

**Fig. 4:**
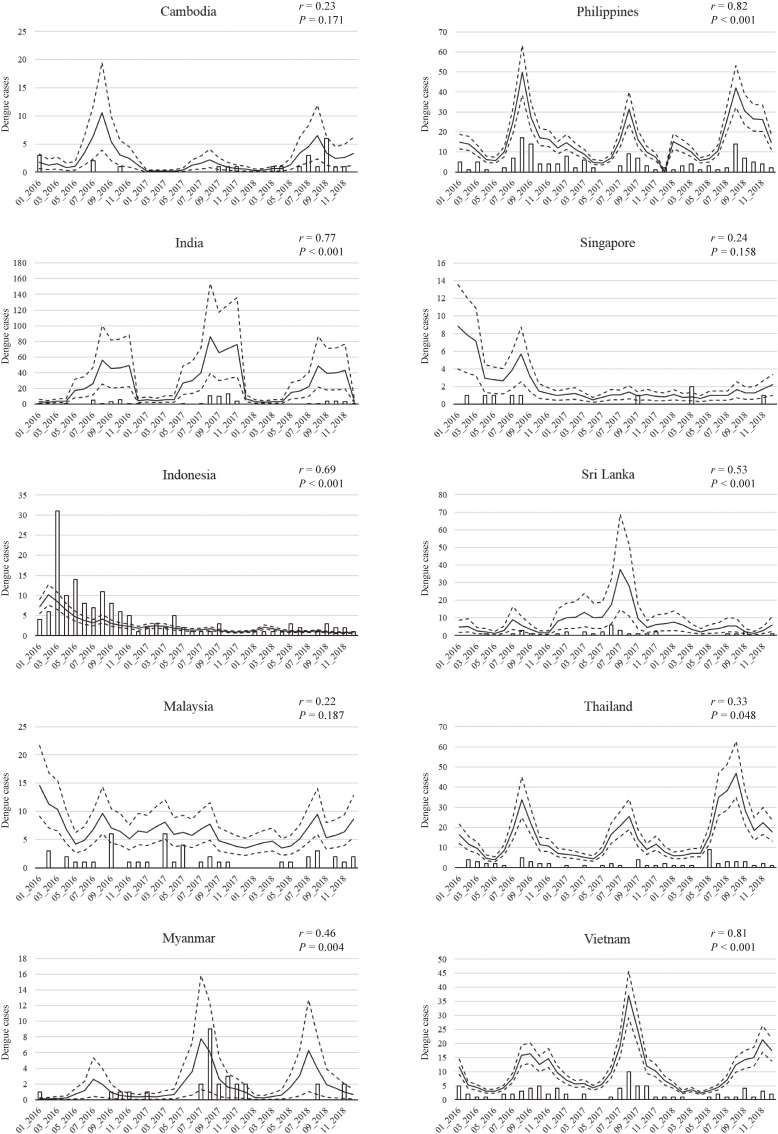
Estimated number of Japanese dengue cases (symptomatic) and the actual number of imported cases reported. The solid line for each country is the median and the dashed lines represent the 95% DI. Vertical bars indicate the imported cases reported in Japan.

We compared the number of dengue symptomatic Japanese cases estimated using this simulation model with the actual number of imported dengue cases reported by medical institutions in Japan by destination country (Fig. 4). The Philippines (*r* = 0.82, *P* < 0.001), Vietnam (*r* = 0.81, *P* < 0.001), and India (*r* = 0.77, *P* < 0.001) showed strong correlations between the estimated number of Japanese dengue symptomatic cases and the number of reported Japanese imported cases. Indonesia (*r* = 0.69, *P* < 0.001), Sri Lanka (*r* = 0.53, *P* < 0.001), and Myanmar (*r* = 0.46, *P* = 0.004) showed moderate correlations. A weak correlation was observed in Thailand (*r* = 0.33, *P* = 0.048). The results for Cambodia, Malaysia, and Singapore were not statistically significant. For Cambodia, Indonesia, and Myanmar, the actual number of imported cases in Japan generally fell within the 95% DI of the estimates.

### Dengue infection risk per year: current estimate and calculated from seroprevalence

**Fig. 5:**
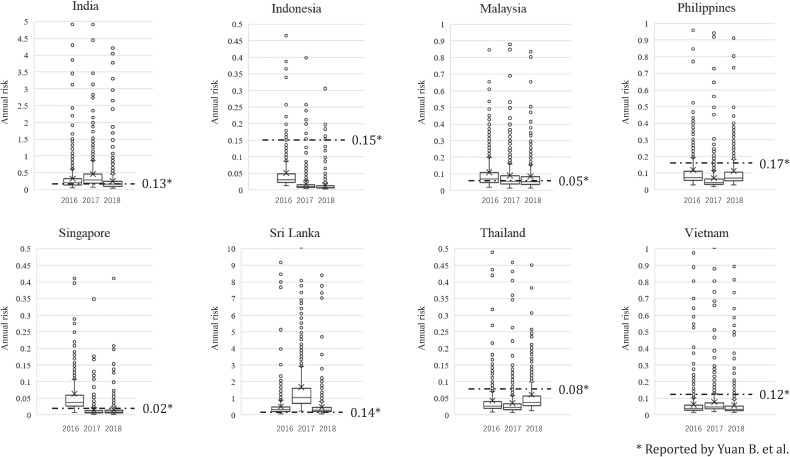
Annual risk of dengue infections estimated in this study and values calculated from seroprevalence rates. The distribution for each year is shown as a box plot. The cross mark in each boxplot indicates the mean value.

Yuan et al. reported annual dengue infection rates calculated from seroprevalence in eight of the ten countries investigated in our study [11]. To confirm the proposed model’s accuracy, the results were compared with the estimates obtained (Fig. 5, Table 2). In 21 of the 24 cases (i.e., 8 countries × 3 years, 2016–2018), the 95% DI of the estimated dengue infection rate included the values calculated from seroprevalence. The only cases in which the annual infection rates calculated from seroprevalence deviated from 95% DI were Indonesia in 2017 and 2018 and Sri Lanka in 2017.

**Table 2:** Annual percentage of dengue infections comparison between this study’s estimates and seroprevalence rate-based calculations.

	**Yuan et al. [** 11 **]** **(95% confidence interval)**	**Proportion of dengue infections by year (95% DI)**		

		**2016**	**2017**	**2018**
India	0.13 (0.12–0.13)	0.20 (0.07–1.05)	0.29 (0.10–1.50)	0.16 (0.05–0.81)
Indonesia	0.15 (0.14–0.17)	0.03 (0.02–0.16)	0.01 (0.01–0.05)*	0.01 (0.00–0.04)*
Malaysia	0.05 (0.05–0.05)	0.07 (0.03–0.36)	0.06 (0.02–0.29)	0.05 (0.02–0.28)
Philippines	0.17 (0.15–0.19)	0.07 (0.04–0.36)	0.04 (0.02–0.21)	0.07 (0.03–0.35)
Singapore	0.02 (0.02–0.02)	0.04 (0.01–0.21)	0.01 (0.00–0.04)	0.01 (0.00–0.05)
Sri Lanka	0.14 (0.13–0.15)	0.32 (0.10–1.79)	1.06 (0.34–5.99)*	0.29 (0.09–1.65)
Thailand	0.08 (0.07–0.08)	0.03 (0.01–0.14)	0.02 (0.01–0.11)	0.04 (0.02–0.19)
Vietnam	0.12 (0.11–0.13)	0.04 (0.02–0.19)	0.05 (0.02–0.24)	0.03 (0.02–0.18)

*The 95% DI of our estimates does not include the annual infection rate calculated from the seroprevalence.

## Discussion

*P_IMPORT_* from all 10 endemic countries in Japan was almost 1.0, and *P_AUTO_* in Japan from June to September was approximately 1.0 between 2016 and 2018. Correlations between our estimated symptomatic Japanese cases and actual imported cases reported in Japan were observed in seven countries. Our estimated annual risks of dengue infection in the eight endemic countries were similar to those reported by Yuan et al. [11].

### Usefulness of the monthly dengue cases reported in endemic countries

In endemic countries, DF is not completely seasonal, and even when it is seasonal, the peak season varies from country to country. To more accurately estimate the infection risk by travelers, such as *P_IMPORT_* and *P_AUTO_*, having enough detail on the infection situation in each country is important. Availability of the number of DF monthly reports in each target country would be useful, but this approach is rather difficult to implement. The data source repositories’ location varied; in some cases, only graphs without numbers were presented.

Currently, no standard approaches to understanding DF seasonality in endemic countries exist. For instance, Salami et al. [48] used DengueMap [49], and the only information they relied on was “whether there was seasonality in each country.” In contrast, Liebig et al. [50] employed information from the International Association for Medical Assistance to Travellers [51] and inferred the seasonal distributions of dengue cases based on information from just two source countries (Brazil and Thailand).

Under these circumstances, unifying the number of monthly reports in endemic countries internationally and making the data more accessible would greatly contribute to modeling studies, even if the diagnostic criteria and medical regimes in each country differ.

### *P_IMPORT_* and *P_AUTO_*

The median *P_IMPORT_* for all and Japanese travelers entering Japan from any SEA/SA countries was 1.0. This means that at least one DENV-infected traveler was estimated to enter Japan every month from any of the ten SEA/SA countries. *P_IMPORT_* among SEA/SA visitors to Japan was lower compared to Japanese because the number of international visitors was approximately 60% (Japanese travelers) between 2016 and 2018, and the susceptibility to DENV was set at one-fifth that of the Japanese.

Lai et al. reported the median monthly import risk from nine SEA countries to more than ten Chinese cities was 0.18 in 2005 and 0.98 in 2015 [14]. In 2015, 9.5 million Chinese returned to China, and 2.5 million SEA country travelers visited China for 12 million. Contrastingly, in our study, the monthly *P_IMPORT_* for all travelers was 1.0, even though the number of Japanese and SEA/SA travelers in 2016 was 7.6 million, less than that reported in China. This is mainly because our *P_IMPORT_* was calculated for Japan as a whole and not for each city within Japan. However, whether each parameter is used appropriately is unknown. Lai et al. reported an infection period of *D* = 10 days (SD 1 day), and the same value was used in our study. Conversely, another report states that “the infection period is from approximately 1.5 days before the onset of symptoms to approximately 5 days after it (about 7 days in total)” [52]. Furthermore, infected individuals may stop going out after the onset of symptoms (i.e., the opportunity for mosquito transmission is reduced) or be less infectious if asymptomatic. We performed sensitivity analyses under two conditions, *D* = 7 days (SD 1 day) and *D* = 2 days (SD 1 day) (Supplementary Figs. 1, 2). The results confirm that a shorter infection period would, to some extent, reduce *P_IMPORT_*.

*P_AUTO_* for June–September was 1.0 for all SEA/SA countries, indicating that *P_AUTO_* is highly dependent on temperature conditions in Japan. Our model used the average temperature in Tokyo as a representative value for Japan. When the average temperature is 22 °C or higher and the mosquito survival rate increases from June to September, autochthonous transmission risk in Japan is sufficiently high for DENV brought from any country.

However, no autochthonous dengue transmission was reported in Japan between 2016 and 2018. *Ae. aegypti* and *Ae. albopictus* is highly adapted to urban and peri-urban areas and breeds in various artificial and natural containers in urban and rural areas [53–55]. The first reason for the low number of reports of autochthonous infection in Japan may be the further urbanization of the country. Improved airtightness of buildings and widespread use of air conditioning reduce the mosquito bites possibility through human-mosquito contact [56]. Second, diagnosing DF is difficult. Clinically, severe symptoms are often present during the second infection, and most first-time infections are asymptomatic [57, 58]. Most Japanese are DENV-naïve and are likely unaware of their infection. Moreover, DF has a high frequency of nonspecific symptoms, such as fever, headache, and myalgia, and the diagnosis is difficult without a confirmed history of travel and mosquito bites. A few mosquito bites during summer in urban Japan are unlikely to be noticed as an illness history, making it difficult to link them to DF diagnosis [59]. Third, *Ae. Albopictus’s* ability to transmit dengue infection may be lower than that of *Ae. aegypti* [60, 61].

The high *P_AUTO_* of 1.0 from June to September is because, as in the case of our *P_IMPORT_*, the infection risk for the entire country was summed up in this study and not broken down by city. Conversely, we calculated the number of mosquitoes per capita as 2 (SD 1), as reported by Lai et al. This value could be much lower in urban areas of Japan. Therefore, a sensitivity analysis was conducted with the number of mosquitoes per capita set at 0.4 (SD 0.2) (Supplementary Figs. 3). The results showed a slight decrease in *P_AUTO_*, but not significant. Therefore, the present simulation results may have been greatly affected by whether the infection risk in the endemic countries was properly calculated.

### Estimated Japanese cases and imported cases reported in Japan

The estimated number of Japanese dengue cases, including asymptomatic patients, in the ten target countries from 2016 to 2018 totaled 16,205. There were 660 imported cases reported in Japan during the same period, with a difference of 24.6 times. Yuan et al. reported that the actual number of dengue cases among Japanese travelers was >20 times compared to imported cases [11], similar to the ratio we obtained. However, in a simulation in China by Lai et al., the ratio of the estimated number of DENV-infected travelers from nine SEA countries to the actual number of imported cases reported in China was 13.5 times [14]. Although the number of reported dengue infections after 2016 was higher globally than before, it has been noted that EF may be overreported during epidemics [13]. Such effects may have led to the difference between our study and Lai et al.

We assumed that the infection risk for travelers was the same as that for residents in endemic countries. However, this is likely to differ significantly from country to country. The ratio of the estimated number of symptomatic dengue cases per month among the Japanese to the actual number of imported cases reported in Japan and the strength of the correlation differed by endemic country. Regarding the number of imported cases reported in Japan, the same diagnostic conditions apply upon return, regardless of the country visited. Therefore, using the number of imported Japanese cases reported in Japan as a benchmark, the difference in infection risk between Japanese travelers and local residents in each country may be estimated. This method will enable more accurate predictions in the future.

The estimated total number of symptomatic Japanese dengue cases visiting 10 SEA/SA countries between 2016 and 2018 was 3,220, of which approximately 20% (n = 660) were diagnosed with dengue infection at a Japanese medical facility after returning to Japan. This gap exists because some Japanese travelers may have been infected in dengue-endemic countries but had recovered when they returned to Japan and had not visited a Japanese medical institution. Dengue infection incubation period is said to be 4–10 days (3–7 days in most cases), the duration of fever is 2–7 days [1, 62], and the average length of stay for Japanese used in this model ranged from 3.3 to 17.4 days. The patient might have visited a hospital after returning to Japan, but the necessary tests for dengue diagnosis were not performed or were overlooked because the symptoms had already resolved. Neumayr et al. found that 64% of imported dengue cases returning to Europe visited a hospital after 7 days of onset (mainly for follow-up or confirmation of diagnosis) [63]. If most Japanese DENV-infected patients (imported cases) who visited a medical institution after returning to Japan are likely to be after the acute phase (7 days after onset), that is, even after the resolution of acute phase symptoms, this may explain the underreporting of the number of imported cases in Japan.

### Annual infection rate estimated in this study and calculated from seroprevalence rate

The annual dengue infection rates reported by Yuan et al. were based on seroprevalence surveys in each SEA country from 2005 to 2016 [11], a different period than ours (2016–2018). Furthermore, the asymptomatic infected patients’ proportion (*μ*) used in our model is highly variable [64, 65], and EF is also expected to change (gradually decline) as medical conditions improve. However, our estimates of annual dengue infection rates and those calculated from the seroprevalence rates were similar in many cases, except for Indonesia in 2016–2018 and Sri Lanka in 2017. The seroprevalence data used by Yuan et al. in Indonesia [66] was collected in urban areas, which may have resulted in a higher calculated infection rate compared to the national average (as we noted earlier, *Ae. aegypti* is more distributed in urban areas). For Sri Lanka in 2017, the number of reported infections during the year was much higher than in other years, so the predicted number of infections is also higher in our simulation (Fig. 4). Considering that seroprevalence measurements are not frequently available, this simulation model, which can be used when monthly numbers of reported dengue infections are available for each SEA/SA country, is a useful alternative for estimating infection risk in cases where recent seroprevalence data are unavailable.

### Limitations

First, we did not consider differences in activities/behaviors at the travel destination because no information was available. Although a detailed analysis is currently difficult, if more detailed data on destinations can be confirmed and made available in the future, the estimation model accuracy can be improved. For example, data on the number of Japanese visitors to Bali in Indonesia are available from JTB data [29]. Myanmar can also distinguish the number of Japanese visitors to the three international airports [33]. Second, the differences in autochthonous transmission in Japan based on the presence or absence of clinical symptoms were not considered. Some reports suggest that even asymptomatic patients may be more infectious to mosquitoes if their dengue viremia levels reach a certain level, as they are more active than patients with clinical symptoms [67]. Because our *P_AUTO_* was summed over the entire country, the autochthonous transmission risk could be explained almost exclusively by temperature; however, the infection severity may need to be considered in the future when each region is considered separately. Third, making direct comparisons among SEA/SA countries is difficult because of the varying criteria for clinical diagnosis and reporting of dengue infections in each country [68]. Fourth, although the risk was estimated only for Japanese and SEA/SA residents in this study, they are not the only actual travelers. Travel routes are much more complex, including residents of other countries passing through SEA/SA on their way to Japan. The third and fourth are difficult to address immediately, but our simulation used the best available data. While this is a future challenge, we expect the model to improve by harmonizing diagnostic criteria across countries and centralizing more detailed traveler data.

## Conclusions

The number of dengue cases from SEA/SA to Japan was estimated using EF, the monthly number of patients with dengue in each endemic area, and the number of Japanese and SEA/SA travelers showed consistent correlations with the actual number of imported cases reported in Japan. However, domestic imported cases may be underreported compared to actual symptomatic cases. The risk of dengue importation into Japan can be high enough, and attention should be paid to autochthonous transmission spread between June and September when mosquitoes are active in Japan. Estimates of seasonal risk variation from each DENV-endemic country can be used to inform preventive and control measures for dengue in Japan.

## Abbreviations

DI: Data interval
DF: Dengue fever
DHF: Dengue hemorrhagic fever
DENV: Dengue virus
EF: Expansion Factor
EIP: Extrinsic incubation period
JNTO: Japan National Tourism Organization
SEA/SA: Southeast and South Asia
SD: Standard deviation

## Supplementary information

**Additional file 1:** **Supplementary Document:** Description of the mathematical model.

**Additional file 2:** **Supplementary Table:** Number of travelers per country.

**Additional file 3:** **Supplementary Figures 1–3:** Sensitivity analyses.

## Declarations

### Availability of data and materials

All data are incorporated into the article.
