# Supplementary material for: Mathematical model estimation of dengue fever transmission risk from Southeast and South Asia into Japan between 2016 and 2018
Source: Environ Health Prev Med. 2023 Sep 9;28:50. doi: 10.1265/ehpm.22-00267 (PMC10495242; doi:10.1265/ehpm.22-00267)
Supplement: Supplementary file 1 — Additional file 1: Supplementary Document: Description of the mathematical model. [file ehpm-28-050-s001.docx]

**Supplementary Document**

**Description of the mathematical model**

**Purpose and description of the model**

This study aimed to measure the seasonal probability of dengue fever introduction from endemic countries by using a framework developed and reported by Lai et al.[1] that integrates air travel, epidemiological, demographical, entomological, and meteorological data.

In our study, it uses a branching process modeling framework assessing the risk of dengue introduction from Southeast and South Asia into Japan by integrated three factors: 1) the risk of a person acquiring the disease in the origin country; 2) the probability of a person traveling to the destination country of interest while infectious, and 3) the likelihood of subsequent local transmission in the destination country. The model considered seasonal and inter-annual risks with respect to the international spread of infectious diseases, as well as the possibility that the relative exposure risk and importation probability in travelers may differ between local residents in endemic areas and residents of non-endemic areas traveling to endemic countries.

**Definition of** $\boldsymbol{P}_{\boldsymbol{IMPORT}}$ **and** $\boldsymbol{P}_{\boldsymbol{AUTO}}$

$P_{IMPORT}$: Probability that at least one dengue virus (DENV)-infected traveler is flying from Southeast and South Asia (SEA/SA) into Japan and is infectious upon arrival in Japan. It was defined as a single-step Poisson process depending on: 1) the risk of infection in travelers during the period of stay in the country with ongoing dengue virus transmission; 2) the probability of non-Japanese residents in SEA/SA traveling into Japan, and the probability of Japanese travelers returning to Japan; and 3) the duration of infection in humans as the length of the intrinsic incubation period for DENV plus the time that a person remains viremic after onset, referring to the period over which an infected person could travel and experience symptomatic disease or transmit DENV to mosquitoes.

$P_{AUTO}$: Monthly probability of autochthonous transmission of at least one person in Japan by DENV brought from SEA/SA. It was defined as the probability in a three-step process: 1) infected airline travelers from each SEA/SA country entering Japan; 2) mosquitoes in Japan acquiring the virus from infected travelers; and 3) those infected mosquitoes infecting at least one other person in Japan. The latter two processes, human-to-mosquito and mosquito-to-human DENV transmission in Japan were characterized as Poisson processes with means of the number of infectious mosquitoes produced per infected human and humans infected per infectious mosquito.

**Dengue importation risk into Japan (**$\boldsymbol{P}_{\boldsymbol{IMPORT}}$**)**

The dengue importation risk in Japan and the autochthonous transmission risk in Japan were calculated by simulation, and the model was based on a report by Lai et al. [1]. The simulation was run 1,000 times.

The actual number of dengue cases, $I_{s,m}$, in each month is estimated using the equation (1).

$I_{s,m}$ = $\frac{I_{s,m}^{R}F_{s}}{\mu}$ (1)

$I_{s,m}^{R}$ is the actual number of reported cases per month $m$ in country $s$. $F_{s}$ is the expanshion factor (EF) in each country, and $\mu$ is the percentage of symptomatic cases. $\mu$ was set at 20% (SD = 10%)[2,3].

Person-days for Japanese travelers, $A_{s,m}$, and locals in SEA/SA countries, $B_{s,m}$, are calculated as in equations (2) and (3).

$A_{s,m}=C_{j}T_{s,m}^{j}$ (2)

$B_{s,m}= F_{m}G_{s,m} - C_{f}T_{s,m}^{f}$ (3)

$T_{s,m}^{j}$ and $T_{s,m}^{f}$ are the numbers of Japanese travelers to SEA/SA countries and SEA/SA travelers to Japan, respectively. $C_{j}$ and $C_{f}$ are the average number of days spent in the SEA/SA country by Japanese tourists and the average number of days spent in Japan by SEA/SA nationals, with SD set at 10%. $F_{m}$ is the number of days in each month and $G_{s,m}$ is the population of the year in country $s$.

The daily prevalence of dengue infection, $E_{s,m}$, is calculated by equation (4).

$E_{s,m}$ = $\frac{I_{s,m}D}{A_{s,m}+ B_{s,m}}$ (4)

$D$ is the period during which a person is infectious and is the sum of the incubation period of DENV and the time the virus remains after the onset of the disease, which is 10 days (SD 1 day)[4].

$\lambda_{s,m}^{j}$ and $\lambda_{s,m}^{f}$ refer to person-days for which Japanese returning to Japan and SEA/SA country travelers coming to Japan are infectious and are calculated as equation (5) or (6).

$\lambda_{s,m}^{j}$ = $E_{s,m}T_{s,m}^{j}R_{s}^{j}C_{j}$ (5)

$\lambda_{s,m}^{f}$ = $E_{s,m}B_{s,m}R_{s}^{f}\frac{T_{s,m}^{f}}{G_{s,m}}$ (6)

$R_{s}^{j}$ and $R_{s}^{f}$ represent the susceptibility to DENV of Japanese and SEA/SA nationals with $R_{s}^{j}$ set to 1 and $R_{s}^{f}$ set to 0.2 (SD 10%)[3].

The risk of at least one DENV-infected travelers visiting Japan, $P_{IMPORT}$, is calculated according to a Poisson distribution using equations (7－10).

$P_{IMPORT\_j}(s,m)$ = 1 − $e^{{－\lambda}_{s,m}^{j}}$ (7)

$P_{IMPORT\_f}(s,m)$ = 1 － $e^{{－\lambda}_{s,m}^{f}}$ (8)

$\lambda_{s,m}$ = $\lambda_{s,m}^{j}$ + $\lambda_{s,m}^{f}$ (9)

$P_{IMPORT}\left( s,m \right)$ = 1 － $e^{-\lambda_{s,m}}$ (10)

**Dengue autochthonous transmission risk in Japan (**$\boldsymbol{P}_{\boldsymbol{AUTO}}$**)**

The relationship between mortality and temperature for *Ae. Aegypti* and *Ae. albopictus* is calculated using the following polynomial equation:[5,6]

$\mu\left( T \right)=0.3967-0.03912T+(2.442\times{10}^{-3})T^{2}-(7.479\times$ ${10}^{-5})T^{3}+(9.298 \times$ ${10}^{-7})T^{4}$ (11)

$T$ is the average temperature of the mosquito's habitat area.

The average lifespan of mosquitoes per month, $L_{m}$, the time until mosquitoes become infectious after biting, the extrinsic incubation period ($EIP$), and the percentage of mosquitoes surviving during the $EIP$, $\gamma_{m}$, are calculated by equations (12－14).

$L_{m}= \frac{1}{\mu(T)}$ (12)

$EIP {=e}^{(\log{EIP}_{28})e^{\beta_{T}(T-28)}}$ (13)

$\gamma_{m}= e^{- \frac{EIP}{L_{m}}}$ (14)

$EIP$ at 28°C (${EIP}_{28}$) is set to 6 days (SD 2 days), with $\beta_{T}=-0.08$ (SD 0.02) as the coefficient related to temperature[7].

The density of mosquitoes per capita per month, $\varphi_{m}$, is calculated using the equation (15).

$\varphi_{m}=\varphi(\frac{L_{m}}{max L})$ (15)

$\varphi$ is the density of mosquitoes under ideal weather conditions, 2 (SD 1), and $max L$ is the largest value of $L_{m}$ in each year.

The number of DENV-infected mosquitoes generated from one infected person, $R_{m}^{HM}$, and the number of people infected by one infected mosquito, $R_{m}^{MH}$, are calculated by equations (16) and (17).

$R_{m}^{HM}= \varphi_{m}\alpha\beta_{HM}V\gamma_{m}$ (16)

$R_{m}^{MH}=\alpha\beta_{MH}L_{m}$ (17)

The rate of mosquito bites per day, $\alpha$, is set at 0.7 (SD 0.05)[5,8], and the probability of infection of a mosquito that bites an infected person, $\beta_{HM}$, and the probability of infection of a person bitten by an infected mosquito, $\beta_{MH}$, are both set at 0.5 (SD 0.1). The number of days, $V$, that an infected person was infectious to mosquitoes is set to four days (SD 1 d)[4].

The risk of dengue transmission by at least one person, $P_{AUTO}$, in Japan is calculated as

$P_{AUTO}\left( s,m \right)=1- e^{\lambda_{s,m}[e^{R_{m}^{HM}\left( e^{-R_{m}^{MH}}-1 \right)}-1]}$ (18)

**The reasons for selecting these parameters in the sensitivity** **analyses**

$$P_{IMPORT}$$

Parameters $\mu$, $D$, $F_{s}$, $C$ and $R_{s}$ were used to calculate $P_{IMPORT}$. Although $\mu$ and $F_{s}$ considered highly variable across years and environmental conditions, there is limited information to determine their validity and how much they should be varied, and we determined that they are not appropriate for use in our sensitivity analysis. As for $R_{s}$, we considered it reasonable to set it at 1.0 for Japanese travelers, who have a greater impact on the simulation results than travelers from endemic countries. On the other hand, $D=7$ days and $D=2$ days were used in the sensitivity analysis because it was reported to be more appropriate for $D=7$ days than the $D=10$ days[9]. We also considered the possibility that $D$ could be even shorter than 7 days. If an infected person shows symptoms, he or she may refrain from going out. Also, if the infected person is asymptomatic, the person may have low levels of the virus in his or her blood. In these cases, we considered that the infection period $D$ could be much shorter, so we also performed a sensitivity analysis under the condition $D=2$ days.

$$P_{AUTO}$$

Parameters $\alpha$, $\beta_{HM}$, $\beta_{MH}$, $V$, $\varphi$, $L_{m}$ and $\beta_{T}$ were used to calculate $P_{AUTO}$. It was difficult to determine the validity of $\alpha$, $\beta_{HM}$, $\beta_{MH}$ and $L_{m}$ since there is little information on them, and they are considered to vary greatly depending on environmental conditions and other factors. $V$ is the human infections period, and we used 4 days as in Lai et al. We considered this value reasonable since there was another similar report[9]. $\beta_{T}$ was already defined by a polynomial equation. Conversely, the number of mosquitoes per capita, $\varphi$, was set to 2.0 in the simulation, but it could be lower in urban areas in Japan, where most people reside. Therefore, $\varphi$ was varied in our sensitivity analysis.

**References**

1. Lai S, Johansson MA, Yin W, Wardrop NA, van Panhuis WG, Wesolowski A, et al. Seasonal and interannual risks of dengue introduction from South-East Asia into China, 2005-2015. PLoS Negl Trop Dis. 2018 Nov 1;12(11):e0006743.

2. Grange L, Simon-Loriere E, Sakuntabhai A, Gresh L, Paul R, Harris E. Epidemiological risk factors associated with high global frequency of inapparent dengue virus infections. Front Immunol. 2014;5:280.

3. Clapham HE, Cummings DAT, Johansson MA. Immune status alters the probability of apparent illness due to dengue virus infection: Evidence from a pooled analysis across multiple cohort and cluster studies. PLoS Negl Trop Dis. 2017 Sep 27;11(9):e0005926.

4. Guzman MG, Harris E. Dengue. Lancet. 2015 Jan 31;385(9966):453–65.

5. Johansson MA, Powers AM, Pesik N, Cohen NJ, Erin Staples J. Nowcasting the spread of Chikungunya Virus in the Americas. PLoS One. 2014 Aug 11;9(8):e104915.

6. Brady OJ, Johansson MA, Guerra CA, Bhatt S, Golding N, Pigott DM, et al. Modelling adult Aedes aegypti and Aedes albopictus survival at different temperatures in laboratory and field settings. Parasit Vectors. 2013;6:351.

7. Chan M, Johansson MA. The Incubation Periods of Dengue Viruses. PLoS One. 2012 Nov 30;7(11):e50972.

8. Scott TW, Amerasinghe PH, Morrison AC, Lorenz LH, Clark GG, Strickman D, et al. Longitudinal Studies of Aedes aegypti (Diptera: Culicidae) in Thailand and Puerto Rico: Blood Feeding Frequency. J Med Entomol. 2000;37(1):89–101.

9. Carrington LB, Simmons CP. Human to mosquito transmission of dengue viruses. Front Immunol. 2014;5:290.
