# Supplementary material for: Mathematical model estimation of dengue fever transmission risk from Southeast and South Asia into Japan between 2016 and 2018
Source: Environ Health Prev Med. 2023 Sep 9;28:50. doi: 10.1265/ehpm.22-00267 (PMC10495242; doi:10.1265/ehpm.22-00267)
Supplement: Supplementary file 3 — Additional file 3: Supplementary Figures 1–3: Sensitivity analyses. [file ehpm-28-050-s003.pptx]

## Slide 1
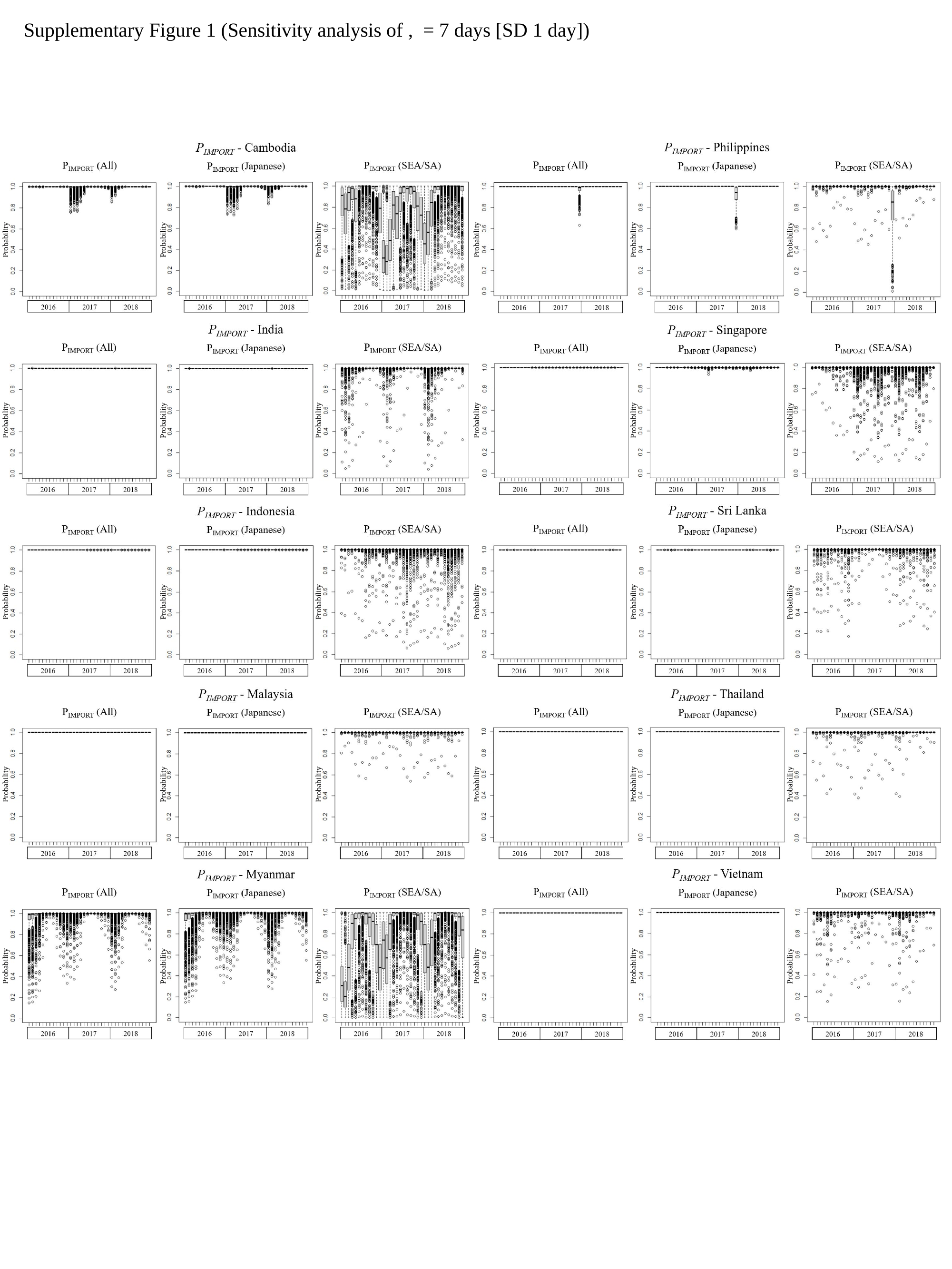

Probability
Probability
Probability
Probability
Probability
Probability
Probability
Probability
Probability
Probability
Probability
Probability
Probability
Probability
Probability
Probability
Probability
Probability
Probability
Probability
Probability
Probability
Probability
Probability
Probability
Probability
Probability
Probability
Probability
Probability

## Slide 2
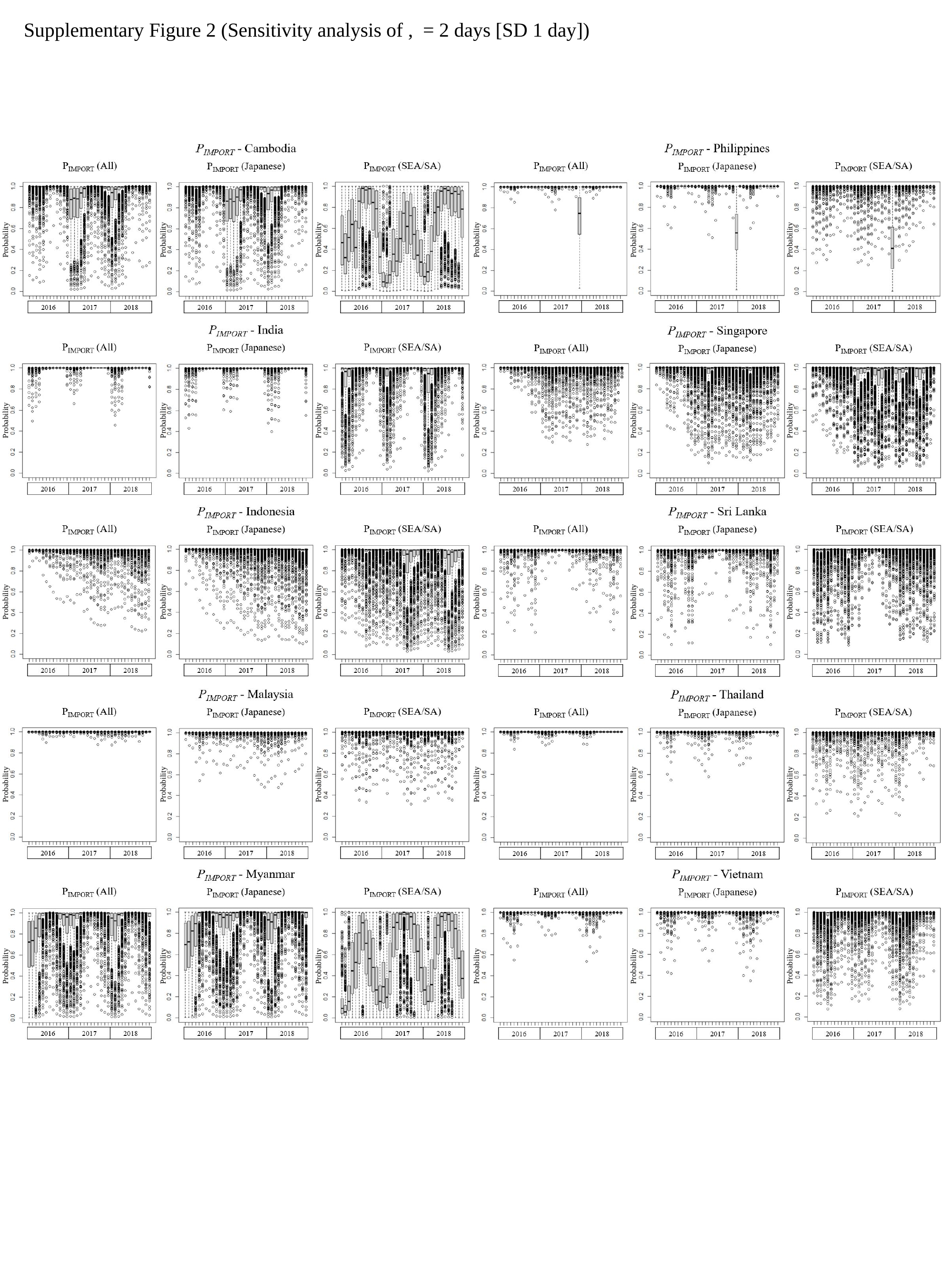

Probability
Probability
Probability
Probability
Probability
Probability
Probability
Probability
Probability
Probability
Probability
Probability
Probability
Probability
Probability
Probability
Probability
Probability
Probability
Probability
Probability
Probability
Probability
Probability
Probability
Probability
Probability
Probability
Probability
Probability

## Slide 3
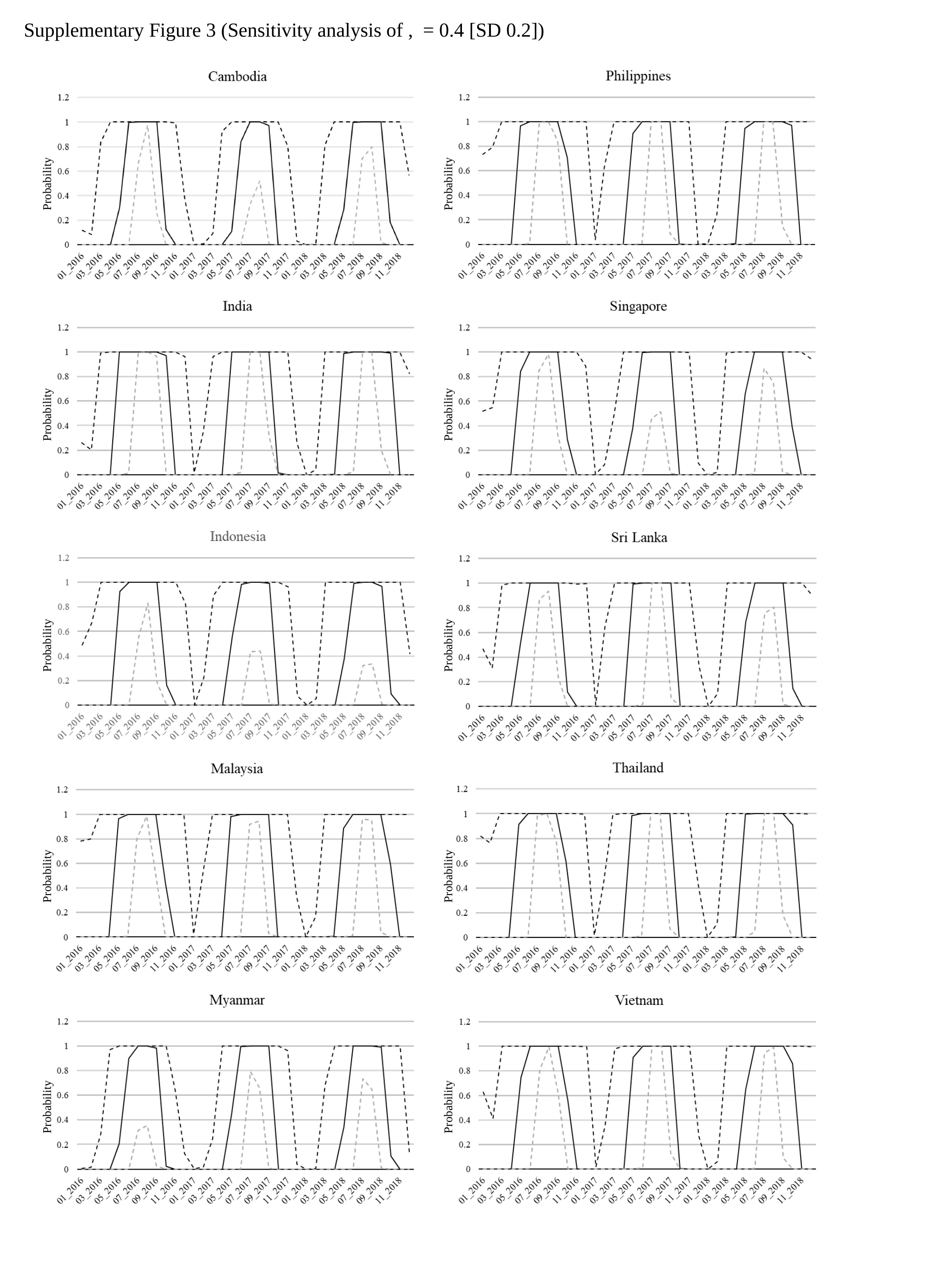

Probability
Probability
Probability
Probability
Probability
Probability
Probability
Probability
Probability
Probability
